# Supplementary material for: Altered Cord Blood Lipid Concentrations Correlate with Birth Weight and Doppler Velocimetry of Fetal Vessels in Human Fetal Growth Restriction Pregnancies
Source: Cells. 2022 Oct 2;11(19):3110. doi: 10.3390/cells11193110 (PMC9562243; doi:10.3390/cells11193110)
Supplement: Supplementary file 1 [file cells-11-03110-s001.zip › Final Supplementary Tables/Suppl Table S4.pdf]

Table S4. Mean eicosanoid concentrations (pmol/mL) measured in umbilical vein plasma.

	SGA Controls (n=12)		FGR (n=7)		
**Eicosanoid**	**Mean / Median**	**95% CI / IQR**	**Mean / Median**	**95% CI / IQR**	**P value**
6-keto-PGF1α	8.311	4.44, 12.18	7.730	3.52, 11.94	0.829
PGF2α	1.308	0.83, 1.79	0.972	0.55, 1.39	0.299
TXB2	3.770	4.77	5.200	11.66	0.34
PGD2	0.654	0.94	0.678	1.03	0.592
PGE2	7.911	9.27	6.081	9.48	0.482
5-HETE	1.969	1.56, 2.38	3.441	1.7, 5.18	**0.022**
12-HETE	4.268	4.8	8.720	65.3	**0.028**
15-HETE	4.584	2.31	4.642	3.09	0.967
5,6-DiHET	0.413	0.32, 0.51	0.498	0.39, 0.61	0.221
8,9-DiHET	3.557	1.4	4.031	4.09	0.482
14,15-DiHET	2.635	2.14, 3.14	3.395	2.49, 4.3	0.08
11,12-DiHET	2.770	1.69	3.834	2.38	0.227
5,6-EET	1.015	0.87, 1.17	1.862	1.4, 2.33	**0.0001**
8,9-EET	0.603	0.49, 0.71	0.959	0.68, 1.24	**0.004**
11,12-EET	0.263	0.17, 0.36	0.684	0.38, 0.99	**0.001**

Mann Whitney nonparametric test performed for non-normally distributed data, presented as median and IQR. Normally distributed data analyzed using unpaired t test, presented as mean and 95% CI. **Bold** indicates statistical significance. Abbreviations: SGA, small for gestational age; FGR, fetal growth restriction; CI, confidence interval; IQR, interquartile range; PG, prostaglandin; TXB, thromboxane; HETE, hydroxyeicosatetraenoic acid; DiHET, dihydroxyeicosatrienoic acid; EET, epoxyeicosatrienoic acid
