# Supplementary material for: Altered Cord Blood Lipid Concentrations Correlate with Birth Weight and Doppler Velocimetry of Fetal Vessels in Human Fetal Growth Restriction Pregnancies
Source: Cells. 2022 Oct 2;11(19):3110. doi: 10.3390/cells11193110 (PMC9562243; doi:10.3390/cells11193110)
Supplement: Supplementary file 1 [file cells-11-03110-s001.zip › Final Supplementary Tables/Suppl Table S5.pdf]

Table S5. Mean nonesterified fatty acid concentrations (μmol/L) measured in umbilical vein plasma.

	SGA Controls (n=12)		FGR (n=7)		
**Free Fatty Acid**	**Median**	**IQR**	**Median**	**IQR**	**P value**
14:0	4.904	3.94	7.619	5.91	0.104
16:1	2.457	5.59	7.586	15.02	0.059
16:0	52.921	35.30	86.109	20.79	**0.022**
18:3	0.072	0.04	0.144	0.07	**0.009**
18:2	17.368	19.59	41.869	28.50	0.056
18:1	28.071	31.36	57.558	46.02	**0.036**
18:0	19.649	9.17	27.206	14.41	**0.022**
20:4	7.521	3.52	9.688	12.35	**0.013**
20:5	0.215	0.19	0.264	0.40	0.217
22:6	3.044	3.40	4.353	2.86	0.142

Mann Whitney nonparametric test performed for non-normally distributed data, presented as median and IQR. **Bold** indicates statistical significance. X:Y nomenclature where X is number of carbon atoms and Y i number of double bonds. Abbreviations: SGA, small for gestational age; FGR, fetal growth restriction; IQR, interquartile range
