# Supplementary material for: Altered Cord Blood Lipid Concentrations Correlate with Birth Weight and Doppler Velocimetry of Fetal Vessels in Human Fetal Growth Restriction Pregnancies
Source: Cells. 2022 Oct 2;11(19):3110. doi: 10.3390/cells11193110 (PMC9562243; doi:10.3390/cells11193110)
Supplement: Supplementary file 1 [file cells-11-03110-s001.zip › Final Supplementary Tables/Suppl Table S7.pdf]

Table S7. Mean phosphatidylcholine concentrations (pmol/mg protein) measured in placenta homogenate.

	SGA Controls (n=12)		FGR (n=8)		
**PC Compound**	**Median**	**IQR**	**Median**	**IQR**	**P value**
16:0_20:4	6255.700	10148.78	4648.450	5741.05	0.427
16:1_20:4	17.650	73.05	4.700	37.93	0.865
O-16:0_20:4	388.700	578.93	285.100	441.03	0.521
O-16:1_20:4	428.550	588.60	277.550	625.03	0.910
18:0_20:4	2582.000	3979.70	2188.300	2830.08	0.384
18:1_20:4	1023.100	2334.63	854.900	1746.95	0.792
18:2_20:4	454.400	978.45	346.000	1035.05	0.792
O-18:0_20:4	196.800	332.90	160.750	248.00	0.473
O-18:1_20:4	329.600	696.20	226.950	592.48	0.734
16:0_20:5	0.000	8.90	0.000	0.38	0.483
O-16:0_20:5	5.300	5.90	3.750	5.10	0.613
O-16:1_20:5	2.300	2.10	4.550	1.63	0.137
18:0_20:5	0.000	0.00	0.000	0.00	0.697
O-18:0_20:5	5.100	6.05	3.200	4.80	0.791
O-18:1_20:5	2.700	3.45	4.000	2.15	0.613
16:0_22:4	76.700	120.58	113.100	174.20	0.507
O-16:0_22:4	23.900	38.83	24.200	42.05	0.867
O-16:1_22:4	15.100	34.48	16.600	30.45	0.748
18:0_22:4	23.150	59.43	49.900	86.13	0.467
O-18:0_22:4	5.350	8.63	8.400	10.05	0.415
O-18:1_22:4	6.600	13.60	6.800	11.13	0.984
16:0_22:5	53.250	107.40	57.600	116.78	0.605
O-16:0_22:5	21.800	20.30	14.450	31.20	0.968
O-16:1_22:5	19.100	19.30	10.400	26.20	0.968
18:0_22:5	9.200	37.55	18.550	41.90	0.552
O-18:0_22:5	7.000	7.20	5.500	7.48	0.794
O-18:1_22:5	9.300	13.90	7.750	18.73	0.987
16:0_22:6	140.200	339.63	109.250	250.88	0.413
O-16:0_22:6	21.050	42.25	15.050	35.40	0.427
O-16:1_22:6	16.600	35.15	14.400	34.75	0.970
18:0_22:6	33.800	135.78	32.950	89.85	0.500
O-18:0_22:6	13.500	22.40	8.000	14.30	0.455
O-18:1_22:6	26.300	38.90	10.800	40.58	0.640

Mann Whitney nonparametric test performed for non-normally distributed data, presented as median and IQR. X_Y nomenclature where X and Y are the fatty acids esterified to either the sn1 or sn2 position. The O denotes an ether linkage. Abbreviations: SGA, small for gestational age; FGR, fetal growth restriction; PC, phosphatidylcholine; IQR, interquartile range
