# Supplementary material for: Altered Cord Blood Lipid Concentrations Correlate with Birth Weight and Doppler Velocimetry of Fetal Vessels in Human Fetal Growth Restriction Pregnancies
Source: Cells. 2022 Oct 2;11(19):3110. doi: 10.3390/cells11193110 (PMC9562243; doi:10.3390/cells11193110)
Supplement: Supplementary file 1 [file cells-11-03110-s001.zip › Final Supplementary Tables/Suppl Table S6.pdf]

Table S6. Mean triglyceride concentrations (pmol/mg protein) measured in placenta homogenate.

	SGA Controls (n=12)		FGR (n=8)		
**TG Compound**	**Median**	**IQR**	**Median**	**IQR**	**P value**
48:1	0.000	3.45	11.800	17.78	**0.008**
48:0	16.750	21.58	35.250	26.38	**0.020**
50:2	94.400	60.10	93.000	103.98	0.305
50:1	121.350	59.08	152.550	131.48	0.057
50:0	6.850	19.20	9.950	31.53	0.294
52:4	6.500	35.28	6.350	83.28	0.721
52:3	153.650	139.03	110.750	282.25	0.792
52:2	185.400	121.93	213.900	231.05	0.270
52:1	35.750	37.05	43.850	42.53	0.296
54:6	6.650	39.85	7.700	62.28	0.898
54:5	31.400	92.35	39.950	142.83	0.717
54:4	21.000	75.55	37.950	117.23	0.388
54:3	57.650	92.58	74.550	74.30	0.343
54:2	2.700	20.50	18.500	31.05	0.115
56:7	15.800	54.83	12.050	76.88	0.831
56:6	7.650	47.33	2.850	71.45	0.831

Mann Whitney nonparametric test performed for non-normally distributed data, presented as median and IQR. **Bold** indicates statistical significance. X:Y nomenclature used where X is total number of carbons on all 3 acyl chains and Y is number of double bonds. Abbreviations: SGA, small for gestational age; FGR, fetal growth restriction; TG, triglyceride; IQR, interquartile range
