# Supplementary material for: Altered Cord Blood Lipid Concentrations Correlate with Birth Weight and Doppler Velocimetry of Fetal Vessels in Human Fetal Growth Restriction Pregnancies
Source: Cells. 2022 Oct 2;11(19):3110. doi: 10.3390/cells11193110 (PMC9562243; doi:10.3390/cells11193110)
Supplement: Supplementary file 1 [file cells-11-03110-s001.zip › Final Supplementary Tables/Suppl Table S2.pdf]

Table S2. Mean phosphatidylcholine concentrations (µmol/L) measured in umbilical vein plasma.

	SGA Controls (n=12)		FGR (n=7)		
**PC Compound**	**Mean / Median**	**95% CI / IQR**	**Mean / Median**	**95% CI / IQR**	**P value**
16:0_20:4	161.126	26.04	175.676	66.71	0.482
16:1_20:4	0.012	0.124	0.404	0.5	**0.034**
O-16:0_20:4	7.646	6.52, 8.77	9.021	6.99, 11.05	0.153
O-16:1_20:4	5.704	4.84, 6.57	6.677	4.9, 8.45	0.213
18:0_20:4	139.918	127.96, 151.88	140.297	109.54, 171.05	0.975
18:1_20:4	10.368	9.33, 11.4	12.277	8.28, 16.27	0.182
18:2_20:4	0.454	0.35, 0.56	0.827	0.17, 1.49	0.096
O-18:0_20:4	6.095	5.33, 6.86	7.591	6.17, 9.01	**0.03**
O-18:1_20:4	5.762	4.94, 6.59	7.388	5.66, 9.11	**0.038**
16:0_20:5	1.836	1.06, 2.62	2.123	0.87, 3.37	0.642
O-16:0_20:5	0.109	0.09, 0.13	0.169	0.09, 0.25	**0.033**
O-16:1_20:5	0.066	0.05, 0.08	0.071	0.02, 0.12	0.773
18:0_20:5	1.077	0.57, 1.59	0.875	0.29, 1.46	0.576
O-18:0_20:5	0.100	0.08, 0.12	0.142	0.05, 0.23	0.171
O-18:1_20:5	0.067	0.05, 0.08	0.096	0.03, 0.16	0.187
16:0_22:4	3.866	3.14, 4.6	3.846	3.06, 4.63	0.968
O-16:0_22:4	1.977	1.59, 2.37	2.233	1.7, 2.77	0.381
O-16:1_22:4	1.110	0.89, 1.33	1.287	0.91, 1.67	0.334
18:0_22:4	3.273	2.74, 3.81	3.355	2.87, 3.84	0.822
O-18:0_22:4	0.476	0.4, 0.55	0.560	0.46, 0.66	0.14
O-18:1_22:4	0.588	0.49, 0.69	0.714	0.57, 0.86	0.108
16:0_22:5	9.227	7.56, 10.9	7.934	5.83, 10.03	0.293
O-16:0_22:5	0.893	0.74, 1.05	0.909	0.78, 1.04	0.876
O-16:1_22:5	0.510	0.43, 0.59	0.586	0.44, 0.74	0.255
18:0_22:5	5.626	4.61, 6.64	4.535	3.19, 5.88	0.158
O-18:0_22:5	0.389	0.32, 0.46	0.446	0.32, 0.57	0.332
O-18:1_22:5	0.437	0.35, 0.52	0.530	0.44, 0.63	0.125
16:0_22:6	38.919	33.75, 44.09	34.427	24.60, 44.25	0.313
O-16:0_22:6	1.063	0.89, 1.24	1.323	1.05, 1.6	0.068
O-16:1_22:6	0.556	0.47, 0.64	0.619	0.45, 0.79	0.406
18:0_22:6	18.558	15.62, 21.49	15.029	9.74, 20.32	0.159
O-18:0_22:6	0.892	0.75, 1.03	1.016	0.78, 1.26	0.279
O-18:1_22:6	0.744	0.63, 0.86	0.788	0.55, 1.03	0.663

Mann Whitney nonparametric test performed for non-normally distributed data, presented as median and IQR. Normally distributed data analyzed using unpaired t test, presented as mean and 95% CI. **Bold** indicates statistical significance. X_Y nomenclature where X and Y are the fatty acids esterified to either the sn1 or sn2 position. The O denotes an ether linkage. Abbreviations: SGA, small for gestational age; FGR, fetal growth restriction; PC, phosphatidylcholine; CI, confidence interval; IQR, interquartile range
