# Supplementary material for: Altered Cord Blood Lipid Concentrations Correlate with Birth Weight and Doppler Velocimetry of Fetal Vessels in Human Fetal Growth Restriction Pregnancies
Source: Cells. 2022 Oct 2;11(19):3110. doi: 10.3390/cells11193110 (PMC9562243; doi:10.3390/cells11193110)
Supplement: Supplementary file 1 [file cells-11-03110-s001.zip › Final Supplementary Tables/Suppl Table S3.pdf]

Table S3. Mean lysophosphatidylcholine concentrations (µmol/L) measured in umbilical vein plasma.

	SGA Controls (n=12)		FGR (n=7)		
**LPC Compound**	**Mean / Median**	**95% CI / IQR**	**Mean / Median**	**95% CI / IQR**	**P value**
16:0-LPC	44.766	38.00, 51.53	34.775	20.87, 48.68	0.108
16:1-LPC	2.867	2.39, 3.34	1.860	0.73, 2.99	**0.038**
18:0-LPC	11.42	3.25	11.762	7.13	0.34
18:1-LPC	12.677	10.63, 14.72	9.564	4.41, 14.72	0.138
18:2-LPC	12.046	10.31, 13.78	9.271	4.59, 13.95	0.135
20:4-LPC	9.300	7.58, 11.02	6.501	2.98, 10.02	0.078
20:5-LPC	0.010	0.03	0.000	0.004	0.08
22:4-LPC	0.096	0.06, 0.13	0.061	0.02, 0.1	0.182
22:5-LPC	0.114	0.07, 0.15	0.049	0.01, 0.09	**0.026**
22:6-LPC	0.400	0.2	0.246	0.214	**0.013**

Mann Whitney nonparametric test performed for non-normally distributed data, presented as median and IQR. Normally distributed data analyzed using unpaired t test, presented as mean and 95% CI. **Bold** indicates statistical significance. X:Y nomenclature where X is number of carbon atoms and Y is number of double bonds in the fatty acid that remains esterified to the glycerol backbone following remodeling of PC compound. Abbreviations: SGA, small for gestational age; FGR, fetal growth restriction; PC, phosphatidylcholine; LPC, lysophosphatidylcholine; CI, confidence interval; IQR, interquartile range
