# Supplementary material for: Altered Cord Blood Lipid Concentrations Correlate with Birth Weight and Doppler Velocimetry of Fetal Vessels in Human Fetal Growth Restriction Pregnancies
Source: Cells. 2022 Oct 2;11(19):3110. doi: 10.3390/cells11193110 (PMC9562243; doi:10.3390/cells11193110)
Supplement: Supplementary file 1 [file cells-11-03110-s001.zip › Final Supplementary Tables/Suppl Table S10.pdf]

Table S10. Mean nonesterified fatty acid concentrations (pmol/mg protein) measured in placenta homogenate.

	SGA Controls (n=12)		FGR (n=8)		
**Free Fatty Acid**	**Mean**	**95% CI**	**Mean**	**95% CI**	**P value**
14:0	57.400	50.97, 63.83	50.904	41.19, 60.62	0.201
16:1	86.999	68.59, 105.41	58.518	35.04, 81.99	**0.043**
16:0	1947.423	1627, 2267	1703.991	1253, 2155	0.316
18:3	4.460	3.54, 5.38	2.739	1.8, 3.67	**0.011**
18:2	616.589	527, 706	561.742	386, 737	0.492
18:1	514.985	432, 598	453.230	297, 609	0.393
18:0	1003.368	880, 1127	920.123	752, 1088	0.367
20:4	3387.849	2800, 3976	3127.627	2204, 4052	0.575
20:5	38.017	29.19, 46.84	30.207	18.77, 41.65	0.231
22:6	267.910	188, 348	218.607	133, 305	0.37

Normally distributed data analyzed using unpaired t test, presented as mean and 95% CI. **Bold** indicates statistical significance. X:Y nomenclature where X is number of carbon atoms and Y i number of double bonds. Abbreviations: SGA, small for gestational age; FGR, fetal growth restriction; CI, confidence interval
