# Supplementary material for: Altered Cord Blood Lipid Concentrations Correlate with Birth Weight and Doppler Velocimetry of Fetal Vessels in Human Fetal Growth Restriction Pregnancies
Source: Cells. 2022 Oct 2;11(19):3110. doi: 10.3390/cells11193110 (PMC9562243; doi:10.3390/cells11193110)
Supplement: Supplementary file 1 [file cells-11-03110-s001.zip › Final Supplementary Tables/Suppl Table S1.pdf]

Table S1. Mean triglyceride concentrations (µmol/L) measured in umbilical vein plasma.

	SGA Controls (n=12)		FGR (n=7)		
**TG Compound**	**Mean / Median**	**95% CI / IQR**	**Mean / Median**	**95% CI / IQR**	**P value**
48:3	0.075	0.29	0.892	1.34	**0.007**
48:2	2.7	1.7	6.17	8.44	**0.02**
48:1	5.522	3.08	9.968	11.02	**0.02**
48:0	2.943	1.54	5.812	3.96	**0.0008**
50:4	0.912	0.88	2.844	2.99	**0.04**
50:3	7.831	4.59	17.586	17.24	**0.005**
50:2	21.287	11.21	40.008	43.2	**0.01**
50:1	20.022	10.41	27.706	21.32	**0.002**
50:0	2.215	1.44	3.348	2.152	0.08
52:5	2.463	1.73, 3.2	5.191	2.14, 8.24	**0.017**
52:4	11.59	8.59, 14.59	28.465	16.28, 40.65	**0.001**
52:3	29.982	21.89, 38.08	68.581	39.83, 97.33	**0.001**
52:2	38.231	25.37, 51.1	72.49	35.02, 109.96	**0.024**
52:1	6.475	4.79, 8.17	11.034	3.42, 18.65	0.09
52:0	1.02	0.83, 1.21	1.273	0.77, 1.78	0.203
54:6	4.891	3.57, 6.21	11.351	6.18, 16.52	**0.002**
54:5	8.828	6.71, 10.95	18.523	11.09, 25.96	**0.002**
54:4	9.58	6.86, 12.30	20.489	12.95, 28.03	**0.001**
54:3	11.343	8.35, 14.34	20.85	13.02, 28.68	**0.006**
54:2	4.243	3.15, 5.33	8.087	3.19, 12.98	**0.031**
54:1	0.163	0.06, 0.27	0.419	-0.11, 0.94	0.154
56:7	4.261	3.07, 5.45	8.003	4.52, 11.49	**0.009**
56:6	2.418	1.47, 3.37	4.807	2.25, 7.36	**0.024**
56:5	0.236	0.10, 0.38	1.086	0.14, 2.03	**0.012**

Mann Whitney nonparametric test performed for non-normally distributed data, presented as median and IQR. Normally distributed data analyzed using unpaired t test, presented as mean and 95% CI. **Bold** indicates statistical significance. X:Y nomenclature used where X is total number of carbons on all 3 acyl chains and Y is number of double bonds. Abbreviations: SGA, small for gestational age; FGR, fetal growth restriction; TG, triglyceride; CI, confidence interval; IQR, interquartile range
