# Supplementary material for: Altered Cord Blood Lipid Concentrations Correlate with Birth Weight and Doppler Velocimetry of Fetal Vessels in Human Fetal Growth Restriction Pregnancies
Source: Cells. 2022 Oct 2;11(19):3110. doi: 10.3390/cells11193110 (PMC9562243; doi:10.3390/cells11193110)
Supplement: Supplementary file 1 [file cells-11-03110-s001.zip › Final Supplementary Tables/Suppl Table S8.pdf]

Table S8. Mean lysophosphatidylcholine concentrations (pmol/mg protein) measured in placenta homogenate.

	SGA Controls (n=12)		FGR (n=8)		
**LPC Compound**	**Mean / Median**	**95% CI / IQR**	**Mean / Median**	**95% CI / IQR**	**P value**
16:0-LPC	248.650	84.13	256.700	64.23	0.401
16:1-LPC	2.483	1.21, 3.75	2.550	1.34, 3.76	0.936
18:0-LPC	102.967	84.07, 121.87	92.975	71.83, 114.12	0.446
18:1-LPC	64.983	52.74, 77.23	62.538	45.01, 80.06	0.791
18:2-LPC	35.742	21.05, 50.44	35.750	12.92, 58.58	0.999
20:4-LPC	25.850	25.28	26.400	30.15	0.827

Mann Whitney nonparametric test performed for non-normally distributed data, presented as median and IQR. Normally distributed data analyzed using unpaired t test, presented as mean and 95% CI. X:Y nomenclature where X is number of carbon atoms and Y is number of double bonds in the fatty acid that remains esterified to the glycerol backbone following remodeling of PC compound. Abbreviations: SGA, small for gestational age; FGR, fetal growth restriction; PC, phosphatidylcholine; LPC, lysophosphatidylcholine; CI, confidence interval; IQR, interquartile range
