# Supplementary material for: Altered Cord Blood Lipid Concentrations Correlate with Birth Weight and Doppler Velocimetry of Fetal Vessels in Human Fetal Growth Restriction Pregnancies
Source: Cells. 2022 Oct 2;11(19):3110. doi: 10.3390/cells11193110 (PMC9562243; doi:10.3390/cells11193110)
Supplement: Supplementary file 1 [file cells-11-03110-s001.zip › Final Supplementary Tables/Suppl Table S9.pdf]

Table S9. Mean eicosanoid concentrations (pmol/mg protein) measured in placenta homogenate.

	SGA Controls (n=11*)		FGR (n=7*)		
**Eicosanoid**	**Mean / Median**	**95% CI / IQR**	**Mean / Median**	**95% CI / IQR**	**P value**
6-keto-PGF1α	0.673	0.4, 0.94	0.542	0.33, 0.75	0.447
PGF2α	0.171	0.11	0.133	0.24	0.93
TXB2	0.012	0.07	0.054	0.16	0.42
PGD2	0.402	0.3, 0.51	0.531	0.24, 0.83	0.263
PGE2	0.346	1.45	2.826	6.49	0.659
5-HETE	5.579	2.03	4.668	3.52	0.536
12-HETE	15.729	19.34	14.114	8.68	0.479
15-HETE	11.386	8.54, 14.23	13.38	8.2, 18.56	0.402
5,6-DiHET	0.203	0.16, 0.25	0.203	0.11, 0.3	0.985
8,9-DiHET	0.202	0.13	0.187	0.05	0.706
14,15-DiHET	0.261	0.18, 0.34	0.245	0.16, 0.33	0.757
11,12-DiHET	0.423	0.18	0.643	0.59	0.479
5,6-EET	4.046	2.04	3.813	2.58	0.659
8,9-EET	3.283	2.2, 4.37	3.123	1.3, 4.95	0.854
11,12-EET	2.232	1.42, 3.05	2.332	0.93, 3.74	0.879
14,15-EET	1.912	0.64	1.629	1.61	0.596

Mann Whitney nonparametric test performed for non-normally distributed data, presented as median and IQR. Normally distributed data analyzed using unpaired t test, presented as mean and 95% CI.
Abbreviations: SGA, small for gestational age; FGR, fetal growth restriction; CI, confidence interval; IQR, interquartile range; PG, prostaglandin; TXB, thromboxane; HETE, hydroxyeicosatetraenoic acid; DiHET, dihydroxyeicosatrienoic acid; EET, epoxyeicosatrienoic acid
*eicosanoid concentrations of one study placenta from each group fell below limits of quantification and were not included in analysis
